# “Exploring knowledge-user experiences in integrated knowledge translation: a biomedical investigation of the causes and consequences of food allergy”

**DOI:** 10.1186/s40900-016-0043-x

**Published:** 2016-09-01

**Authors:** Jenna Dixon, Susan J. Elliott, Ann E. Clarke

**Affiliations:** 1 Department of Geography and Environmental Management, University of Waterloo, 200 University Avenue West, Waterloo, ON N2L 3G1 Canada; 2 Department of Medicine, University of Calgary, 2500 University Drive NW, Calgary, AB T2N 1N4 Canada

**Keywords:** Integrated knowledge translation, Knowledge creation, Collaborative, Food allergy

## Abstract

**Plain English summary:**

Food allergy is a serious public health problem in Canada and other high-income countries, as it is potentially life threatening and severely impacts the quality of life for individuals and their families. Yet, many questions still remain as to its origins and determinants, and the best practices for treatment. Formed to tackle these very questions, the GET-FACTS research study centers on a novel concept in biomedical research: in order to make this science useful, knowledge creation must include meaningful interactions with knowledge-users. With this, knowledge-users are present at every stage of the research and are crucial, central and equal contributors. This study reflects on the early part of that journey from the perspective of the knowledge-users. We conducted interviews with all non-scientist members of the GET-FACTS steering committee, representing Canadian organizations that deal with patient advocacy and policy with regards to food allergy. Steering committee members had a clear sense that scientists and knowledge-users are equally responsible for putting knowledge into action and the importance of consulting and integrating knowledge-users throughout research. They also have high expectations for the GET-FACTS integrated process; that this model of doing science will create better scientists (e.g. improve communication skills) and make the scientific output more useful and relevant. Our work highlights both the unique contributions that knowledge-users can offer to knowledge creation as well as the challenges of trying to unify members from such different communities (policy/advocacy and biomedical science). There remains a real need to develop more touch points and opportunities for collaboration if true integration is to be achieved. Despite the obstacles, this model can help change the way knowledge is created in the biomedical world.

**Abstract:**

ᅟ

**Background:**

Despite the burden of food allergic disease many questions remain as to its origins, determinants and best practices for treatment. Formed to tackle these very questions, the GET-FACTS (Genetics, Environment and Therapies: Food Allergy Clinical Tolerance Studies) research study centers around a novel concept in biomedical research: in order to make this science useful, knowledge creation must include meaningful interactions with knowledge-users, known as Integrated Knowledge Translation (IKT). In IKT, knowledge-users are present at every stage of the research and are crucial, central and equal contributors. This paper contributes to this exciting form of research by reflecting on the beginning of that journey from the perspective of the knowledge-users.

**Methods:**

Semi structured in-depth interviews were conducted in year 2 of the 5 year GET-FACTS project with all (*n* = 9) non-scientist members of the GET-FACTS steering committee, representing Canadian organizations that deal with patient advocacy and policy with regards to food allergy. Transcripts were coded and organized by themes developed both deductively and inductively.

**Results:**

Steering committee members indicated a clear sense that scientists and knowledge-users are equally responsible for the translation of knowledge into action and the importance of consulting and integrating knowledge-users throughout research. Overall, these knowledge-users have very high expectations for the GET-FACTS IKT process; they feel that this model of doing science will create better scientists (e.g. improve communication skills) and make the resulting science more useful and relevant; indeed, they reported that this model of knowledge creation can be paradigm shifting.

**Conclusions:**

This study highlights both the unique contributions that knowledge-users can offer to knowledge creation as well as the challenges of trying to unify members from such different communities (policy/advocacy and biomedical science). While our steering committee has a strong conceptual grasp on IKT and vision for their contributions, execution is not without challenges. There remains a real need to develop more touch points and opportunities for collaboration if true integration is to be achieved. Despite the obstacles, the GET-FACTS IKT model represents a new approach to knowledge creation in Canadian biomedical research and can help foster a culture of openness to participant involvement.

## Background

Food allergy is a serious and potentially life threatening condition that has received increasing attention in Western society. Due to its unpredictable nature, food allergic individuals and their families live with a constant sense of risk, striving to balance physical safety with social wellbeing [1–4]. One in 13 Canadians, or 7.5 % of the population, self report at least one food allergy [5] and there is speculation that rates are on the rise. Despite the burden of food allergic disease [6], many questions remain as to its origins, determinants and best practices for treatment. The GET-FACTS (Genetics, Environment and Therapies: Food Allergy Clinical Tolerance Studies) research study was funded for five years to tackle these questions.

In the context of this pressing public health burden there is a need for research that fulfills the priorities and needs of knowledge-users (that is, individuals who are likely to be able to use research results to make informed decisions about health policies, programs and/or practices [7]) *and* provides an effective means to communicate those messages; the challenge is how to make that happen. This is not a problem unique to food allergy research. Indeed, most health research projects must at some point reflect on their contribution to the wider public and the means for which these contributions will be made available. In traditional research this reflection often takes place after the study has been completed and in venues not accessible to the general public (e.g. research conferences, journal publications). There is, however, a growing movement for knowledge-user engagement in the research process [8–11]. This movement recognizes that knowledge is a journey, not an end product and meaningful input from knowledge-users is important at all stages of this journey [12]. The GET-FACTS study challenges the traditional approach to biomedical research by having knowledge-users as equal partners on the knowledge journey. This paper contributes knowledge translation and knowledge co-production literature by reflecting on that journey from the perspective of the knowledge-users.

### Understanding knowledge translation

In a quest to ensure science is useful, much attention has been paid to strategies to bridge the gap between knowledge and action. Putting knowledge to use (or into ‘action’) is a process that goes by many names. While McKibbon and colleagues [13] found over 100 terms for the process (e.g. implementation science, research utilization), in Canadian health research it is referred to as Knowledge Translation (KT). The knowledge journey can be conceptualized by the Knowledge to Action Framework (‘the KTA Framework’) [14, 15]. The framework recognizes two distinct but equal components: Knowledge Creation (knowledge inquiry, synthesis and the creation of products or tools) and the Action Cycle (activities leading to the implementation or application of knowledge). Ideally, both components occur simultaneously, with a give-and-take throughout the research process. However, comprehensive application of the KTA Framework is rare [16]. Most often, knowledge is pushed out into the public domain after the research is completed and knowledge-users are left to piece together its meaning, importance and potential application.

Over the last decade there has been increasing criticism of the end-of-grant approach, both in Canadian health research [17–19] and beyond [20]. Drawing on the experiences and theoretical constructs of the participatory research literature [21] and recognition of the importance of incorporating lay perspectives to tackle contemporary health problems [22–24], Canadian health researchers [14, 25, 26] and the Canadian Institutes of Health Research [7, 27, 28] have begun advocating strongly for and supporting a more inclusive, knowledge-user driven research agenda. In contrast to end-of-grant KT, the involvement of knowledge-users through the entire research journey is referred to as *Integrated Knowledge Translation (IKT*) [7, 21]. In IKT, knowledge-users are present at every stage of the research and are crucial, central and equal contributors. With tremendous potential to be much more complex than traditional forms of research, the IKT model is appealing to researchers and knowledge-users for the promise that researchers will tackle scientific questions with relevance to knowledge-user needs, have a fresh perspective to conduct their analysis and ultimately have an engaged, knowledgeable and ready audience to disseminate results [25, 26]. That is, having users drive the research agenda is one of the best predictors of seeing findings applied [29].

Despite the potential advantages an IKT model, examples of IKT in biomedical research studies are scarce. Instead, studies that engage with knowledge-users tend to only do this at specific points of the research (e.g. either to help with participant recruitment or to disseminate findings after the research is complete) and have a clinical or public health focus [9, 16]. There are few examples in the literature in which true knowledge-users (not simply other health care personnel) are engaged in a biomedical study throughout the entire research journey.

While IKT-specific research is at a “nascent” stage [21] we can draw on the lessons and insights from other initiatives in the co-production of knowledge, specifically noting that others have found that knowledge-users can contribute to research at many stages along the knowledge journey and their contribution can vary widely. Ross and colleagues [30], for example, found that health system managers’ involvement in research could range from formal (not informed about or actively involved in the research process, only provides legitimacy for the research) to responsive (responds to researchers efforts to engage, provides ideas and tactical advice, helps with data collection, results interpretation and knowledge dissemination) to integral (engaged as significant partner, helps shape research process and outcome, research conceptualization, formulation and execution of knowledge transfer strategies). The degree of knowledge-user integration into research has similarly been explored by others, such as Arnstein’s ladder of citizen power [31] or Tritter and McCallum’s more nuanced model that acknowledges diversity in knowledge contributions [32]. Oliver and colleagues [20] provide a framework for public involvement in research that not only addresses the degree to which the knowledge-user is involved (in this case termed ‘consultation, collaboration and lay control’) but also the context to that involvement. That is, are they involved as individuals or as members of organized groups and is it at the invitation of the research programme or in response to action by the lay public?

Ideally, knowledge-users involved in an IKT project should find themselves at the more integrated end of the various spectrums or ladders described above. Yet we know that knowledge-user involvement in research is not without obstacles. Many have described knowledge-users and scientists as coming from two separate vantage points with little common ground and even less common language. The suggestion is that integration of these communities is an unnatural fit and cooperative efforts are formidable [33, 34]. For instance, a feeling of ‘tokenism,’ or the belief by scientists that knowledge-users cannot understand or are not interested in the actual research, can decrease scientist’s willingness to draw on and engage with their partners [35]. Even with all parties fully engaged and willing to participate, different goals and expectations of project outcomes can undermine cooperative tasks [36–38]. Gagliardi et al. [38] note that knowledge-users’ lack of skill or understanding of IKT and poor attitudes towards the research scientists remain significant barriers in IKT studies published to date. Moreover, the very nature of basic or “discovery” research may be harder for knowledge-users to participate in due to its focus on ‘hard’ science and lack of touch points for ‘real’ people [37].

It is in this context that GET-FACTS was designed and implemented within an IKT model. Noting the potential for challenges in engaging knowledge-users in the research journey and yet simultaneously having few concrete examples of IKT within a large-scale biomedical study, we sought to explore the perceptions and experiences of the GET-FACTS knowledge-users, herein referred to as steering committee members, early on this knowledge journey. While we are studying all the GET-FACTS participants (both scientists and steering committee members; a similar investigation of the research scientists is the subject of a campaign paper), this study focuses on knowledge-users, exploring the steering committee’s perceptions of participation at year 2 of 5 in the GET-FACTS IKT project.

### The GET-FACTS project

GET-FACTS is a five-year biomedical research project funded by the Canadian national health granting council. This program of research, led by a coalition of researchers from nine universities across Canada, is designed to investigate food allergy origins, causes and treatment. GET-FACTS is relatively unique among Canadian biomedical research studies in that the KT component functions equally and simultaneously with the core biomedical research right from the initial stages. Of the four pillars of research, a knowledge-user driven research agenda through IKT stands equal to research on genetic determinants of food allergy and tolerance, environmental impact on functional and immunological tolerance to foods, and novel biomarkers to assess allergy and tolerance.

A key element to the KT component is the establishment of an knowledge-user steering committee used to identify issues and help prioritize the research agenda as well as identify the KT opportunities and potential outcomes associated with the project. As the GET-FACTS proposal was originally being formed, 9 representatives from Canadian food allergy policy and advocacy were invited to join the committee based on prior strong relationship with the researchers through related research activities, 6 of whom confirmed and participated in drafting the proposal. Representatives were envisioned to provide the perspectives and priorities of their organizations on food allergy in Canada *and* contribute individual experience and insight where applicable. After the proposal was successful, the KT lead reached out again to representatives to confirm their participation. The top priority of the initial steering committee meeting in March 2014 was to determine other representatives that should be at the table.

One of the primary deliverables of the steering committee over the first 18 months was the development of a Terms of References to guide the functions of the committee and to formalize the project deliverables. Using a consensus decision making model, the group ultimately decided that the steering committee should consist of a maximum of 20 individuals, broken down to 1) a maximum of 5 representatives from the GET-FACTS scientists (both biomedical scientists and social scientists), a maximum of 5 representatives from the policy realm (federal, provincial and municipal/local/regional level governance), and a maximum of 10 representatives from patient oriented and not for profit organizations. The Terms of Reference also notes that members are invited to be a part of the steering committee based on their affiliation with relevant groups/research organizations and in the event that the member must leave the steering committee, another person from that organization will be invited to fill their seat at the table. Under the general principle of keeping the committee comprehensive yet manageable in size, new members can be proposed to the steering committee by any member in good standing at any time with relevant documentation and justification. Any proposed membership will be taken to the full steering committee for affirmation.

Together, members of the steering committee represent a variety of faces of ‘knowledge-users’. For example, members from patient advocacy groups speak to the rest of the committee about how they use research findings and the challenges their organizations face trying to interpret and decode newly published research when the public is contacting them for guidance. These members also speak from personal experiences as parents of children with food allergy. As well, the biomedical GET-FACTS scientist on the committee speaks both as a primary researcher and a practicing physician, and one of the patient advocacy representatives speaks both for her organization and based on her perspective as a practicing dietician.

The steering committee meets in person once or twice annually, with the GET-FACTS researchers as a whole and exclusively as a steering committee, and remains in contact through email exchanges and teleconference calls. The overarching vision of GET-FACTS is to have the steering committee meet regularly with scientists from all research projects under the GET-FACTS umbrella and freely exchange questions and ideas. With this, the steering committee can ask questions, spark research ideas and act as a sounding board for scientists along the knowledge creation journey. While steering committee members have pledged to keep all emerging scientific findings confidential until they are published, their time spent on the committee can help them to communicate back to their organizations the current state of scientific knowledge on food allergies and provide a more comprehensive interpretation of emerging scientific studies. Working alongside the science creation, steering committee members may, in theory, develop a better grasp of the science and later be called upon as a quality source for knowledge dissemination.

At the time of data collection the steering committee had two significant meetings with the GET-FACTS scientists. The first at the inaugural meeting in March 2014 was with the GET-FACTS Principle Investigator, who provided an overview of the entire project, the state of knowledge on food allergy and spent much time interacting with steering committee members to answer any of their questions and to explore potential roles the committee could play in the research going forward. The second major interaction with the scientists occurred October 2014 at the project’s annual meeting. All the theme leads came to the steering committee meeting, presented to the committee an in-depth overview of the science informing their work and provided an update on the state of the project and challenges they were facing. These interactions provided a venue for discussions not normally had between scientist and knowledge-user (for example, why does the analysis of genetic data take so long?).

All members of GET-FACTS (both steering committee and research scientists) have agreed to participate regularly to participate in IKT and to reflect on their IKT experiences. Steering committee members complete feed-back forms after every in-person meeting and both scientists and steering committee members are interviewed by the KT leads regularly through the life of the project. Amongst many advantages, these methods provide the KT arm regular feedback and the opportunity to shift direction when and if necessary, given the exploratory nature of this initiative. For the GET-FACTS IKT model to be successful, all members must be engaged and willing to participate. One of the salient questions going into this research was: how prepared and receptive are steering committee members to bridge the worlds between knowledge-users and research science?

## Methods

This paper focuses on the perspectives of the GET-FACTS steering committee on integration of knowledge-users into the research process (IKT). Semi structured in-depth interviews were utilized in order to provide space and time to explore narratives and experiences of the participants in regards to KT broadly and this project specifically. After receiving ethics approval from the University of Waterloo Office of Research Ethics all nine non-scientist members of the GET-FACTS steering committee were invited and agreed to participate in an interview.

Participants were individually interviewed by phone between March and May 2015. Interviews lasted between 37 and 51 min and were digitally audio-recorded with permission. All participants were assured that their responses would remain anonymous and any published material would be carefully screened so as not to give away the identity of the speaker. Participants were also reminded of their right to decline answering any of the interview questions. This was especially important in this context, given that all potential participants were known to each other and known to others outside the research project (i.e. named members of the GET-FACTS steering committee). The interview questions were informed by the research objectives, probing i) participant background and previous experience working in collaborative research; ii) participant reflections of knowledge translation broadly; and, iii) participant reflections on the GET-FACTS model of IKT.

### Analysis

Audio files were transcribed verbatim and, after a thorough review of the literature, a thematic analysis ensued. A thematic analysis is the search for notable “themes” which are important for describing the phenomenon in question and involves reviewing data to explore both the research goals and any emergent issues. This method allows for pattern recognition within the data, which is carefully read and re-read throughout the process [39, 40]. Pattern recognition in our study occurred both deductively (based on the research objectives and interview guides) and inductively (themes emerging from the interview transcripts) [40].

The first author, who conducted all interviews, reviewed all transcripts and from this created an initial thematic set of codes to identify relevant data, broadly sorted into themes and sub-themes [41]. This initial set was then used independently by the first and second authors for a detailed review and pilot coding of three randomly selected transcripts. The authors then met to discuss their coding and noted any differences or missing codes. The code set was amended to these changes and using QSR International’s NVivo for Mac 10.2 the theme code set was then used to code all the transcripts. To enhance consistency and reliability, the first author led all steps of the coding and analysis process and the second author provided feedback and participated in interpretation of the findings [42].

## Results

Results are organized around three sections: participant backgrounds and previous experiences, perceptions regarding KT and reflections on the GET-FACTS model of IKT. Each theme is discussed in turn below, with key findings punctuated by participants’ voices through anonymous quotations.

### Participant backgrounds

Participants in this study represent a varied array of experience with research and research studies. Four out of the nine members of the GET-FACTS steering committee hold a PhD in the social sciences; one member is a Registered Dietician; and all members hold senior positions within their respective organizations. Every participant reported that accessing current scientific information was important to the work of his or her organization. While seven participants had personally been involved in a research project prior to GET-FACTS, all nine reported that their organizations had previously contributed, in some form, to conducting research. This involvement was cursory for some (for example helping researchers re-work questionnaires so that the language was understandable by their research participants) while others (notably the public health organizations) contributed to designing and conducting research as part of their mandate. All nine participants reported that KT was a central component to the work of their organization.

### Participant perspectives on knowledge translation

All participants had heard of and used the term “Knowledge Translation” prior to its discussion in the interview. When participants were asked what KT meant to them, the most frequent response (7 participants, 10 mentions) emphasized making information useable to the knowledge-user. That is, not just presenting the information, but tailoring it with the needs of the knowledge-user in mind:

*You really try to be careful about the difference between, say, knowledge exchange and [KT]. Exchange is finding ways to share that [information], but translation is actually finding a way to put it into terms and in a context that means it will be used. (Participant 004)*

Participants also emphasized that decoding language and making it understandable to knowledge-users was an important aspect of KT (6 participants, 9 mentions). In the context of their work with food allergy this would mean taking scientific jargon and repackaging it in “everyday” language so that the public could make use of it.

*… it’s almost like you’re taking one language and translating it into a different language; the language being science and the translation ending in common and useful terms. (Participant 005)*

Participant 004 humorously referred to this aspect of KT as “the quest to find the secret decoder ring” in order to act as a broker of KT; to illustrate:

*The problem is scientists are so specialized they don’t know how to speak broadly sometimes. [KT] means overcoming that. So I started researching what the medical terms and everything else meant, and [eventually] I thought, okay, I think I get what she’s saying. But I had to wade deep into that science jargon. It took me a month of writing out notes, figuring out what all these long words meant. But this scientist really believed in her work, so I told myself… I have to figure out how to tell people why this is a good thing. (Participant 001)*

Two thirds of participants (6 participants, 8 mentions) described aspects of IKT when asked to define KT. That is, engaging with knowledge-user groups before and during the data collection was a vital component of KT:

*It means engaging with all relevant stakeholders to better understand the context surrounding a situation. To understand the knowledge and evidence that’s available, processes that are used to make decisions, the potential gaps, how gaps could be filled, what evidence is missing. It’s trying to get a better sense of the entire situation, where the gaps are and how the gaps can be filled. (Participant 007)*

However, while there was general agreement that knowledge translation meant engaging with knowledge-users, only two of these participants explicitly stated that the research should then be guided by the knowledge-user needs.

Many of the participants noted that while they understood the concept of KT, the term “Knowledge Translation” was not what they would use on a regular basis. Four participants said their organization used another term such as ‘knowledge mobilization’ to denote the same concept. Two members of the committee also pointed out the ambiguity of terms such as “Knowledge Translation”. They argued that the ‘siloed’ usage of different terms to convey the same or similar ideas could create more confusion and decrease the exchange of ideas across disciplines. Both of these participants also said that a focus on ambiguous and specialized terms, such as KT, actually created a barrier to the public from engaging with the information, ironically the opposite of what KT is supposed to do:

*We don’t really use [the term] [KT], we like to call it the ‘science policy interface’. It’s easy to get caught up in terminology… I find that in common day language and talking to people, they don’t really quite understand it and usually have to ask ‘what does that really mean’? (Participant 006)*

When asked what groups they thought should be responsible for conducting KT, participants pointed to a variety of actors. Most frequently cited were policy makers and/or the government (8 participants, 10 mentions) and scientists/researchers (8 participants, 10 mentions). Seven participants (9 mentions) suggested that it was the responsibility of stakeholder groups to conduct KT. Others mentioned with much less frequency included industry (2 participants), public health units (1 participant, 1 mention), health professionals (1 participant, 1 mention) and the public (1 participant, 1 mention). Two-thirds (6 participants, 12 mentions) of participants explicitly argued that KT was a shared responsibility amongst the different actors mentioned above. One participant reflects:

*I think if it’s seen as a collective responsibility and is adhered to in a process through the acceptance of multiple participants, then we’ll have a better outcome, I’m sure. (Participant 002)*

Thus, participants made a link between the successes of the KT to the engagement of multiple actors in a variety of roles.

Participants also spoke to their impressions of scientists’ engagement with KT activities. While two participants said that they had not had enough contact with scientists to be able to comment, four of the nine participants (8 mentions) said that, broadly, their sense was scientists were not excited or engaged with KT activities:

*My experience has been that scientists feel it’s not their responsibility; they just want to be able to do the research that they do. (Participant 002)*

Three participants noted that there was a large variability in “scientists” when reflecting on this, and while some are very engaged, others are not. Two participants (2 mentions) pointed out that there are many scientists that would like to become more involved in KT activities, but they face large obstacles in making that happen; the demands of conducting research and publishing consume most of their time and are the priorities of their research institutions:

*From what I’ve seen, they’re all fully in favour of it and want their knowledge to get out there but are pretty entrenched and focused in their research world and can’t do it all. (Participant 006)*

Thus, these participants expressed that while scientists support KT, it is lower on the priority list and fails to materialize.

### GET-FACTS model of IKT

At the time of the interviews, the GET-FACTS steering committee had been active for approximately 13 months, though some individual participants joined after that point. After this year of activity, participants were asked to reflect on the GET-FACTS steering committee IKT model, which was followed by probing for both perceived strengths and challenges going forward. One sentiment was strongly echoed by all participants: it is a model that other research projects need to be adopting. As participant 004 notes, “as a model, it’s where we need to go.”

Four of the participants (4 mentions) expressed that they viewed the GET-FACTS IKT model as a definitive and distinctive change to the way research has traditionally been conducted:

*I think if the model plays out, it could be really powerful… if we can really find a way to positively influence the way work is done, even the way that things are published and things like that, just by getting scientists to think about the end-users all the way through. I think we can really improve their ability to be full serve. Their ability to conceive of and do the research and analyze the findings… (Participant 004)*

This participant speaks to the potential “power” of the model, and the hope that it will be able to shape scientists’ work through each step of research and dissemination.

Over half of those interviewed (5 participants, 7 mentions) expressed the sentiment that it was “too early” to be able to comment on the GET-FACTS IKT model. These participants said that they were still figuring out how the model would work and that the project was still evolving:

*I think for me, it’s too early for me to say. I’m still getting a feel for some of this, as we go along. So, I’m not quite sure yet. (Participant 003)*

Therefore, they said, it was too early to speculate on the model’s connection to the research scientists or knowledge-users:

*Whether [the steering committee’s contributions are] being communicated to the scientific research group, or whether or not they are having or will have any impact on the scientists and getting the research out to the in the real world, the kids and their families… I have no idea if that’s happening, and I think it’s kind of too early at this point for it to have happened. (Participant 009)*

All participants spoke generally of advantages in approaching research in the manner of GET-FACTS. The most commonly suggested strength of the GET-FACTS IKT model was that it could increase the quality and accuracy of knowledge dissemination (7 participants, 7 mentions). Six participants (6 mentions) noted that it could potentially teach scientists how to communicate with lay audiences, a benefit that would ripple beyond the immediate study into scientists’ future projects and other realms of work. As one participant notes, by having scientists talk about their research with non-scientists they will grow to be better communicators:

*I think it makes them more comfortable with the lay-person, who is not in their field. Because if they only talk to people in their field they get away with jargons and acronyms because everybody has the same base of knowledge, right? Whereas a lay-person might not. (Participant 001)*

Others saw another advantage of the model being the capacity to encourage scientists to focus on topics relevant to knowledge-users (5 participants, 6 mentions). For example, one participant proposed that scientists should be aware of the population statistics that patient advocacy groups are greatly in need of, and could potentially frame future research to address these gaps. Further, when research focuses on topics knowledge-users are concerned about, scientists’ efforts become more valuable:

*I think it’s a win-win for everyone. I think that scientists will be able to do a little less wheel spinning and maybe target more on areas that are of major concern. (Participant 003)*

This finding parallels another suggested benefit; the research stemming from this model will be more readily used by knowledge-users (3 participants, 3 mentions):

*Rather than [going] the more traditional model where the researcher just comes up with their research question and their theory, and then they come up with research that may or may not be useful… it’s another research paper to add to the pile with possibly mediocre design and irrelevant statistics and nothing that really changes anything. It’s almost like a make-work project. If you go the other way around and you say, okay we want to improve the life of an allergic kid, [we can ask:] ‘what are the things that are affecting his or her health and the parents’ ability to cope?’ And so [when we] work backwards the advocacy piece will be stronger because you’re having research that’s directly linked to the issues that the child is facing and then it trickles down to the governmental level. The government will then be in a position to provide information that can allow groups to advocate more effectively for the patient. (Participant 005)*

Participants suggested that the GET-FACTS IKT model would provide stakeholders with a greater appreciation and understanding of the research behind the findings (2 participants, 2 mentions), while simultaneously providing scientists with a fresh perspective and ignite new ideas towards the research (4 participant, 4 mentions):

*Sometimes it can even spark different ideas. I really think it’s a win-win situation for everyone involved in the sense of just getting a better understanding of the science, the practise and how each can inform each other. (Participant 007)*

Yet, the participants were also very candid that there were potential challenges the GET-FACTS IKT model may have to confront. Many of the challenges participants suggested involved the dynamics of the steering committee. For instance, many said it would be challenging keeping all members of the steering committee motivated and engaged (including “managing expectations”) (5 participants, 6 mentions) and keeping the steering committee and its meetings organized and focused (5 participants, 6 mentions):

*[The greatest challenge will] involve the steering committee. What are some ways to keep them engaged and motivated? [We will have] to provide some evidence that what we’re doing and what we’re contributing is influencing the science and evidence. […] Providing some concrete examples of how the outcomes of the meetings or other mechanisms were directly heard. (Participant 007)I think [the challenges could be] keeping folks clear on what the focus is and what the limitations of the expectations of the contribution are… (Participant 002)*

The steering committee may also have to confront members’ own biases (3 participants, 3 mentions), especially if some of the results emerging from GET-FACTS were not to the member’s/organization’s liking (3 participants, 4 mentions).

Other potential challenges related to the relationship between steering committee members and the GET-FACTS scientists. Some suggested that the greatest challenge would be overcoming the knowledge gap that exists between stakeholders and scientists (3 participants, 6 mentions). This related strongly to another noted challenge; after a year of existence, some steering committee members still did not feel knowledgeable or connected to the GET-FACTS science (3 participants, 3 mentions), expressing a need for more regular touch points with the science and with the scientists.

When participants were asked to reflect on what in five years “success” or “failure” would look like for the project, a clear pattern emerged. Most participants felt that the success or failure of the GET-FACTS project extended beyond its contributions to food allergy knowledge, though this too was important. Success would be a concrete resource for others to draw on (such as a publication or conference) (5 participants, 6 mentions) and the replication of the model in future research projects (7 participants, 10 mentions). Conversely, participants said failure would be if the GET-FACTS IKT model was not made available for other researchers to learn from (6 participants, 10 mentions) and if the GET-FACTS approach did not influence the conduct of future research projects (7 participants, 7 mentions):

*Failure would be that we all agree that this has been interesting and we can point to some things that we’ve done, and then we all go back to doing things the way we always did them. (Participant 005)*

Participants also expressed that the success or failure of the project related to its contribution to the food allergy knowledge-users. Four participants (4 mentions) expressed success as being a higher quality of life for knowledge-users, such as children with food allergy and their families. Others said success would be an increased amount of information on food allergy made available to the public (3 participants, 3 mentions) and a general increase in the public’s awareness about food allergy (3 participants, 3 mentions). On the other hand, participants said that failure would be if there was no measureable impact from the steering committee (9 participants, 9 mentions), the scientific information from the GET-FACTS project is not well disseminated and is never received by knowledge-users (5 participants, 5 mentions) or if there is a lack of accuracy or credibility in the information that emerges from the project (1 participant, 1 mention). Participants reported (8 participants, 13 mentions) that measuring this impact would be very important for the project to gauge its contribution:

*How do we measure change? How do we know whether people’s awareness has increased, whether their behaviour differs and, most importantly, has their empathy towards food allergy changed? (Participant 002)*

## Discussion and conclusion

Food allergy is a serious public health issue in Canada and beyond for which there are still many unanswered questions surrounding its origins and best practices for treatment. In an endeavour to have science better meet the needs of food allergy knowledge-users, the GET-FACTS project has created a steering committee to represent the perspective and voices of the knowledge-users. The regularized engagement of GET-FACTS scientists with the steering committee throughout the knowledge creation journey represents an innovative approach to KT in biomedical research. Indeed, IKT has rarely, if ever, been executed in such contexts [38]. As we began this journey many questions emerged as to the readiness for both scientists and knowledge-users to engage. This paper explores the perspective of knowledge-users, our steering committee, on their need for IKT and their experiences to date with the GET-FACTS model. While still early knowledge journey (beginning of year 2 of 5) the results of this study speak to the potential and vision for such a model going forward.

These results indicate varying understandings of KT and IKT, but a clear sense from these respondents that scientists and knowledge-users are equally responsible for the translation of knowledge into action. These knowledge-users represent a population that is constantly consuming, interpreting and trying to apply emerging scientific research to the benefit of their constituents: individuals and families affected by the growing epidemic of food allergies. Working with research findings is a part of their daily activities. They have had varying degrees of exposure to the knowledge journey, but their current roles as active members in knowledge creation are new. Participants emphasized that KT is *not* just about dissemination. To them it was about decoding language and repackaging information to have meaning for specific contexts and users. This vantage is obviously informed by their work, which calls for such activities on a daily basis: these participants are on the front lines of food allergy and their work demands making information useful and applicable for policy makers and families dealing with food allergy.

When discussing KT broadly, participants often made reference to the importance of consulting and integrating knowledge-users throughout research. This suggests that they understand KT beyond the traditional linear model (end-of-grant) and have a very good grasp on the IKT model (which stands in contrast to findings from a forthcoming compainion paper investigating responses from the scientists on GET-FACTS) [39]. Participants also noted that, broadly, they saw a gap that exists between scientists and knowledge-users. From their experience, while scientists may have good intentions and may genuinely want to participate in KT, there are barriers to overcome – foremost of this was the use of language. To our participants, how one talks about science, and therefore how one understands science, can be just as important as the scientific findings themselves. While the gap between creators and users of knowledge is well acknowledged in the literature [33, 34] our research underscores the challenges of connecting these communities, even with the bridges created through an IKT approach.

This finding came into focus when discussing the GET-FACTS model of IKT specifically. Participants felt somewhat disconnected from the activities of the scientists. Though the group had presentations from the theme leads to give an overview of the GET-FACTS science, our knowledge-users expressed a sense of waiting for the “real” interaction to occur. This was a red flag for our project and challenged the very purpose of an IKT approach.

By definition, an IKT project should be highly integrated. For example if an IKT project were to be mapped on to Ross and colleagues’ experiences with involving decision-makers in health research [30] knowledge-users should tick all the components of the “integral partner” category (engaged, involvement in decision making activates, helping to shape research). Likewise if mapped on to Oliver and colleagues’ framework for public involvement in health services research [20] an IKT project should register as having a high degree of both public *and* scientist engagement, with quality interactions between the two groups. Yet, our results show that despite its design, GET-FACTS has not yet achieved this level of integration.

One explanation for this may be seen in other IKT studies [35, 36], which have found that the integration of such varying groups of people can be difficult because everyone comes to the table with different backgrounds, different expectations and goals and different conceptions of what the project will look like. As these results bear, this is not a challenge that the GET-FACTS project has been immune to. However, because we are checking in with our members as the project unfolds, this gives us the opportunity to shift rudder midstream. This work tells us that their needs to be extra effort given to overcome these gaps and the creation of more touch points between our steering committee of knowledge-users and scientists. This has begun through a regular webinar series and a joint working group focused on the development of a Performance Measurement Framework for the IKT process within GET-FACTS. With this, GET-FACTS steering committee members and scientists on are working together to create a tool to communicate about the IKT element of the project to internal and external stakeholders, identify outcomes and associated measures for IKT for the GET-FACTS project and develop a model and process for integrated KT for biomedical research more broadly (to be completed September 2016).

Though most of the steering committee members were involved in the GET-FACTS project from conception, reaching the idealized level of integration with the scientists has been slow moving. There are two main factors contributing to this. First, as GET-FACTS scientists are spread out across Canada (in five different time zones) in-person meetings are rare and time is always at a premium. As such, the steering committee’s interaction with the scientists was less frequent and more formal than envisioned. Second, having a co-production of knowledge model (that is, an IKT model) is new ground for Canadian biomedical research and we found that by consequence everything took more time than anticipated. For instance, the creation of the steering committee’s Terms of References took multiple drafts, teleconferences and meetings and was finally finished 18 months into the start of the project. Likewise, creating an expectation of steering committee feedback with the scientists was not an instantaneous process. It took time to ease scientists into this novel concept. By result it is not surprising that the steering committee members find themselves waiting for the real interaction to begin for the GET-FACTS IKT and our project is now, in part through the webinars and the performance measurement framework, trying to play catch-up creating the time and space for genuine interactions to occur and knowledge to be co-produced. Future studies can learn from the challenges experienced by GET-FACTS.

Overall, these knowledge-users have very high expectations for the GET-FACTS IKT process; they feel that this model of doing science will create better scientists (especially scientists with better communication skills) and make the resulting science more useful and relevant. They also reported feeling that – as a result of this model – knowledge users will be better ‘scientists’; that is, they will understand better the research process (how knowledge is created). This perspective from members of the steering committee is one that that the research supports; lay involvement can indeed provide an advantage to health research [22, 24]. Perhaps the most interesting finding was that participants also felt that the GET-FACTS IKT model could fundamentally invert the process of biomedical research. That is, by working backwards and starting with a *problem based focus* (e.g. the allergic child) as opposed to a hypothesis, participants said this would strengthen their role in advocacy for their constituents – Canadians affected by food allergy.

It is perhaps not surprising to reflect on the finding that comprehensive application of the KTA Framework – knowledge creation and knowledge application [14] – is rarely executed [16]. At the onset of this research we knew that an IKT approach to biomedical research would be challenging [37]; even more so with true knowledge-users as partners. But it was also extremely important to the project to have the voices and perspectives of the knowledge-users come to the fore [26]. The GET-FACTS team’s goal was to understand these challenges and adapt the project as it unfolds. This is why it is so noteworthy that these knowledge-users have incredibly lofty goals for this IKT process vis-à-vis markers of success. That is, despite the obstacles ahead, they feel this project actually has the potential to change the scientific paradigm – to change the way research is done. The GET-FACTS scientists have a major challenge in front of them if they are not to disappoint.

## Abbreviations

GET-FACTS: Genetics, environment and therapies: food allergy clinical tolerance studies
IKT: Integrated knowledge translation
KT: Knowledge translation
KTA Framework: Knowledge to action framework
